# Comparison of LABORAS with static incapacitance testing for assessing spontaneous pain behaviour in surgically-induced murine osteoarthritis

**DOI:** 10.1016/j.ocarto.2020.100101

**Published:** 2020-09-28

**Authors:** Isabell S. von Loga, Jadwiga Miotla-Zarebska, Yi-Shu Huang, Richard Williams, Luke Jostins, Tonia L. Vincent

**Affiliations:** a Centre for Osteoarthritis Pathogenesis Versus Arthritis, Kennedy Institute of Rheumatology, University of Oxford, Roosevelt Drive, Oxford, UK; b Kennedy Institute of Rheumatology, University of Oxford, Roosevelt Drive, Oxford, UK

**Keywords:** Pain, Osteoarthritis, Linton incapacitance tester, LABORAS, Mouse

## Abstract

**Objective:**

Evoked responses following mechanical or thermal stimulation are typically used to assess pain behaviour in murine osteoarthritis (OA). However, there is no consensus on how best to measure spontaneous pain behaviour.

**Method:**

OA by partial meniscectomy (PMX), or sham surgery was performed in 10-week old C57BL/6 male mice. Collagen-induced arthritis (CIA) was induced in 10 week old DBA1 male mice. Spontaneous pain behaviour, either at the time of active inflammatory disease (CIA), or over the 12 weeks after induction of OA, was assessed by static incapacitance testing (measuring percentage of weight placed through each hindlimb), and Laboratory Animal Behaviour Observation Registration and Analysis System (LABORAS) (translating cage vibrations of singly house animals into specific activities). Data were analysed by repeated measures two way ANOVA with post hoc testing comparing experimental groups with either sham operated or naïve controls.

**Results:**

By incapacitance testing, two phases of painful behaviour were evident after PMX: a transient, post-operative phase, which resolved within one week, and a late OA pain phase starting 8 weeks post surgery and reaching statistical significance at week 12 (95% CI: sham 89.51–98.19, PMX 76.18–98.16). LABORAS, was able to detect pain behaviour in mice with CIA, but no statistically significant pain behaviour was observed in OA mice either post operatively (once analgesia had been controlled for) or at any later time points for any activity compared with the sham group.

**Conclusion:**

Static incapacitance testing is superior to LABORAS for measuring spontaneous pain behaviour in surgically induced murine OA.

## 1 Introduction

Pain in human osteoarthritis (OA) is the main presenting symptom, with more than 75% of patients experiencing symptoms of pain daily [1]. Given these numbers, understanding the pathogenesis of pain and its subsequent treatment are a major clinical need. Generally, pain can be categorized as either allodynia or hyperalgesia. Allodynia is defined as pain from a stimulus that normally does not elicit a painful response, and hyperalgesia is defined as a reduced threshold of pain from a usually painful stimulus. Both can be measured by evoked pain responses, which can be mechanical or thermal, such as pressure, pinprick, touch (all mechanical) as well as cold and heat (thermal). Both allodynia and hyperalgesia can be maintained and enhanced through central sensitization, where central changes contribute to a maladaptive response to the peripheral painful trigger [2]**.** Measuring pain in human OA is difficult and subjective but there are multiple validated semi-quantitative and qualitative tools, such as the WOMAC score for function [3], the KOOS-PS for knee OA [4], visual analogue scores for pain [5], and the PAIN-DETECT questionnaire for central sensitization [6]. Experimental tools and quantitative sensory testing (QST), which are designed to measure specific modalities such as hyperalgesia and allodynia at sites of and distal to injury are also used [7].

Measuring pain in mouse models of OA is also challenging but there are advantages because disease can be induced unilaterally. Historically, inflammatory models of OA have been shown to elicit strong pain behaviours and in recent years there has been a significant push to validate pain assessments in surgical models of OA that are regarded as more relevant to human disease [8]. There are several ways to induce OA surgically. Destabilization of the medial meniscus (DMM) is a well-established model yielding robust and reproducible disease [9]. Partial meniscectomy (PMX), where part of the medial meniscus is removed, is a slightly more severe model of OA [10].

A number of pain assessments in pre-clinical models have been validated and can be broadly classified into those that are evoked responses and those that are measures of spontaneous pain behaviour [11,12]. Evoked pain responses in OA are usually recorded as the time to withdrawal following a given stimulus at the joint or a site distal to the joint (usually paw). These include mechanical stimulation using von Frey filament testing [13,14], thermal sensitivity using Hargreaves test [15] or a hot/cold plate [14], hind limb withdrawal upon pressure [14,16] or vocalization tests upon joint squeezing [14]. Spontaneous pain responses, where the animal's behaviour is observed and behavioural differences are recorded may be a better reflection of clinically relevant patient pain. These include gait analysis [17], incapacitance testing [9,18,19], and behavioural recording such as using the Laboratory Animal Behaviour Observation Registration and Analysis System (LABORAS) [9,13,20]. The choice of which measure to use is frequently determined by reproducibility within a specific laboratory, and as such, different patterns of pain behaviour are obtained by different groups using different methodologies.

Malfait and colleagues have detected mechanical allodynia after DMM surgery by von Frey filaments [13]. This starts in the immediate post-operative phase and persists in the DMM group compared with sham-operated animals. Another group measured an additional second phase associated with hyperalgesia and allodynia 10 weeks after PMX surgery using a variety of pain measures including von Frey filaments, paw pressure, cold sensitivity and knee compressions [14]. The pain measures by this group also detect an early post-operative phase of differing length (usually resolving within 3 weeks) using paw pressure and knee compressions [14]. In our laboratory, we have measured both the early phase as well as a late phase using static incapacitance testing, a measure that assesses spontaneous rather than evoked pain [9]. In this test, the animal is placed in a chamber that forces it to distribute its weight through the hind limbs. Each hind paw is placed on a separate weighing scale and pain can be inferred by a shift in weight from the diseased side onto the non-diseased side. Both early and late phases of pain behaviour assessed in this way were associated with elevated levels of nerve growth factor (NGF) expression in the joint [9,21,22].

Bias is a particular problem in pain studies especially as they are largely based on subjective behavioural observations. A failure to be blinded to treatment status, time after treatment/disease induction, and side (right or left) of disease can contribute to this problem. Whilst blinding to treatment should be paramount, blinding to time and disease side is often difficult to achieve. Other factors can also impact on behavioural outcome measures such as time of day of testing, environment of testing, person performing test, and other inducers of mouse stress [21].

LABORAS offers a way of minimising bias and allows minimal handling. LABORAS assesses activity levels of 7 different types of spontaneous behaviours based on specific movements from the animals, which are detected by a vibration-sensitive platform and translated as a specific movement by the LABORAS software. It is a useful tool for measuring spontaneous behaviour due to its full automation and with experimental parameters set beforehand. Our group was the first to publish the use of LABORAS in surgically induced OA [9]. A small number of other groups have published on this methodology subsequently [13,20]. In all three papers published to date, different modalities were used as a demonstration of pain behaviour. The lack of consensus encouraged us to undertake a detailed and thorough data collection and analysis of LABORAS to test its robustness and to aim to provide a useful reference for the OA pain community. We compared it with our laboratory gold standard – static incapacitance testing.

## 2 Methods

Animals: All surgeries were performed according to the procedures approved by the UK Home Office (Animals Scientific Procedures Act 1986) and the guidelines issued by the International Association for the Study of Pain. Mice were kept in approved animal-care facilities and were housed 5 per cage in standard individually ventilated cages, maintained with a 12 h light/dark cycle at an ambient temperature of 21°C. Animals were fed a certified mouse diet (RM3 from Special Dietary Systems, Essex, UK) and water *ad libitum*. Sham and PMX operated animals were randomly assigned by our animal surgeon at time of surgery and were mixed throughout cages. Different groups of mice were used for each behavioural test. Some animals were unoperated (naïve). The total number of mice used for this study was 112 (40 PMX, 35 sham, 12 CIA, 25 naïve).

Surgical joint destabilization: 10 week-old male C57BL6 mice (Charles River, UK) were randomised to undergo surgical destabilization by partial meniscectomyor sham surgery as previously described [14]. Weights of all animals were taken (mean 32.4 (95% CI 31.2, 33.6)). Briefly, animals were placed under general anaesthesia by inhalation of Isoflurane (Vetpharma, Leeds, UK); 3% induction, 1.5–2% maintenance in 1.5–2 L/min O_2_. 0.3 mg/ml buprenorphine (Vetergesic Alstoe Animal Health, UK) was administered subcutaneously. For the sham operation, the knee joint was opened to expose the meniscotibial ligament, then closed with sutures. For PMX surgery, the meniscotibial ligament was transected and approximately 1 mm of the medial meniscus removed.

CIA induction: 10-week old male DBA1 mice had arthritis induced by immunization with type II collagen in adjuvant, as previously described [22]. Paw thickness, measured by calibrated micrometer, of all four limbs was used to assess onset of disease [22]. All immunised animals (n = 12) developed arthritis. Only animals with hind paw involvement were included in the pain behaviour assessments (n = 10). All 10 had 2 joint arthritis apart from 1 animal with 3 joint involvement. Pain assessments were performed within 5 days of arthritis onset.

Static incapacitance testing: Static weight bearing measurements were performed using the Linton Incapacitance Tester (Linton Instrumentation, Norfolk, UK), as previously described [9,23]. Briefly, mice were first acclimatised to the chamber during training sessions on two separate occasions over two weeks prior to experimental measurements. Mice were manoeuvred inside the chamber to stand with one hindlimb on each weighing scale. Once position was deemed satisfactory by the operator, weight measurements were taken over a 1 s interval for at least three consecutive measurements. Results were expressed as the percentage of weight transmitted through the operated compared with the contralateral limb. One female observer (I.v.L.) performed the measurements and was blinded to the treatment status of the mice until the end of the behavioural tests.

LABORAS: LABORAS allows for the measurement of mouse activity in an undisturbed environment overnight [24]. Mice were housed singly, with 4 platforms in use at any one time. All mice were measured at the same interval after surgery. Food and water were given *ad libitum*. LABORAS picked up vibrations of animal movement and converted these into behaviour classifications, specifically “climbing”, “locomotion”, “inactivity”, “rearing”, “grooming”, “drinking”, and “eating”. Measurements, except where indicated, started at 15:00, and recorded for 18 h until 9:00 the next day. Analysis was subsequently broken down into 1 h intervals or summed over longer intervals. The dark cycle in the institute was from 19:00 until 7:00.

Statistical analyses: Data are expressed as mean ± standard error of the mean (SEM) or 95% confidence intervals were analysed using GraphPad Prism 8.0 (GraphPad Software, San Diego, CA). Outcome variables were the continuous outputs from either Linton or LABORAS. The independent variables were categorical and either time, treatment or activity. For behavioural comparisons of two groups only and without a repeated-measures timecourse, a *t*-test with Bonferroni multiple comparisons test was used. For behavioural comparisons of three groups and without a repeated-measures timecourse, an ordinary two-way ANOVA with Tukey multiple comparisons test was applied. For any timecourse experiment, a repeated-measures two-way ANOVA with a Bonferroni test for multiple comparisons was used. In case of missing timepoints, a mixed model with a Bonferroni multiple comparisons test was used instead of a repeated-measures two-way ANOVA. This mixed-effects analysis was pre-defined by Prism 8.0. Corrections were related to comparisons displayed within each graph. There were two statistical assumptions: Assumption 1 was that there is homogeneity of the variance and assumption 2 was that the residuals were normally distributed. We tested these formally in Prism 8.0 by visually analysing QQ plots, homoscedasticity plots, and residual plots. In most cases, QQ plots demonstrated normal distribution of data. In several cases, the assumption of homogeneity of variance was not met and in these instances data were either transformed (sqrt) to improve residuals and a repeated-measures 2-way ANOVA was performed or where the transformation still did not meet assumptions, non-repeated t tests with a Bonferroni post hoc test for multiple comparisons was performed. Functional regression analysis was carried out in R (v3.5.1) using the packages fda (v2.4.8) and fda. ucs (v1.5.0) [25]. Time courses were modelled using a combination of Fourier basis functions, with the number of basis functions and the roughness penalty (lambda) chosen by cross validation. Statistical significance was assessed using the maximum F statistic, with a null distribution generated by 1000 permutations. Sample size for two sample t tests of LABORAS data (80% power at p < 0.05) was calculated in advance using a Cohen's d taken from “Climbing Activity” (the largest effect size) from our previous publication [9].

Missing data: There were two instances of missing data in this study: (i) Due to logistical complications of taking overnight recordings, the timings of collection had to change slightly over the course of the study meaning that we are missing some 0600–0900 data, where a mixed-model statistical analysis has been used instead. (ii) In week 12 LABORAS (Fig. 7), data collection was disrupted by a computer update at 0300 affecting 0300–0900 data for four mice (2 for each group).

## 3 Results

### 3.1 Painful behaviour measured by static incapacitance testing

**Fig. 1:**
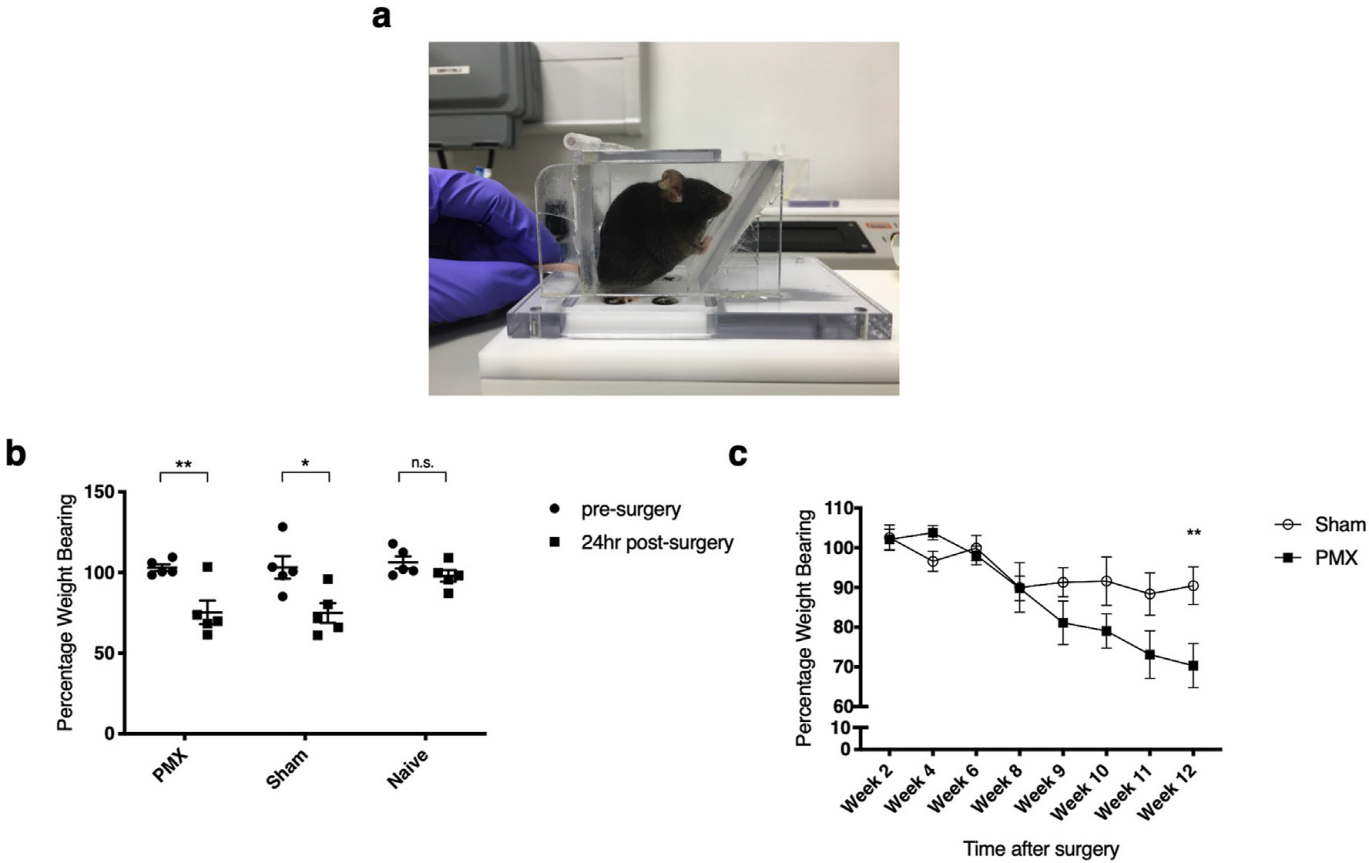
Linton incapacitance testing after OA surgery. (a) Position of animals assessed by Linton incapacitance testing. Animals were lightly restrained to prevent escape by holding the tail with minimal force (b) Weight distribution as measured by Linton incapacitance testing 24 h after surgery (n = 5/group) (lower 95% confidence interval (CI) of mean: pre surgery = 99.53, 24 h post surgery = 50.06; upper 95% CI of mean: pre surgery = 108.8, 24 h post surgery = 115.5). Unpaired *t*-test with significances after Bonferroni multiple comparisons test used. (c) Weight bearing results over the course of OA after PMX or sham surgery. Sham n = 10, PMX n = 15. Repeated measures two-way ANOVA with Bonferroni multiple comparisons test used after transformation of data (square root) to fit statistical assumptions (lower 95% CI of mean: sham = 89.51, PMX = 76.18; upper 95% CI of mean: sham = 98.19, PMX = 98.16. Estimate of difference for week 12 = 6.02). For (b) and (c) SEM shown. ∗p < 0.05. ∗∗p < 0.01. n. s., non-significant.

Spontaneous pain responses for 12 weeks following PMX were measured by static incapacitance testing (Fig. 1a). Post-operative and late OA pain phases were demonstrated. Asymmetrical weight distribution of the hind limbs was recorded 24 h post-surgery and again starting from 8 weeks post PMX surgery (Fig. 1b and c). At 12 weeks the estimate of difference was 6.02 (95% CI: sham 89.51–98.19, PMX 76.18–98.16.

### 3.2 Activity assessment of naive mice by LABORAS over 24 h

**Fig. 2:**
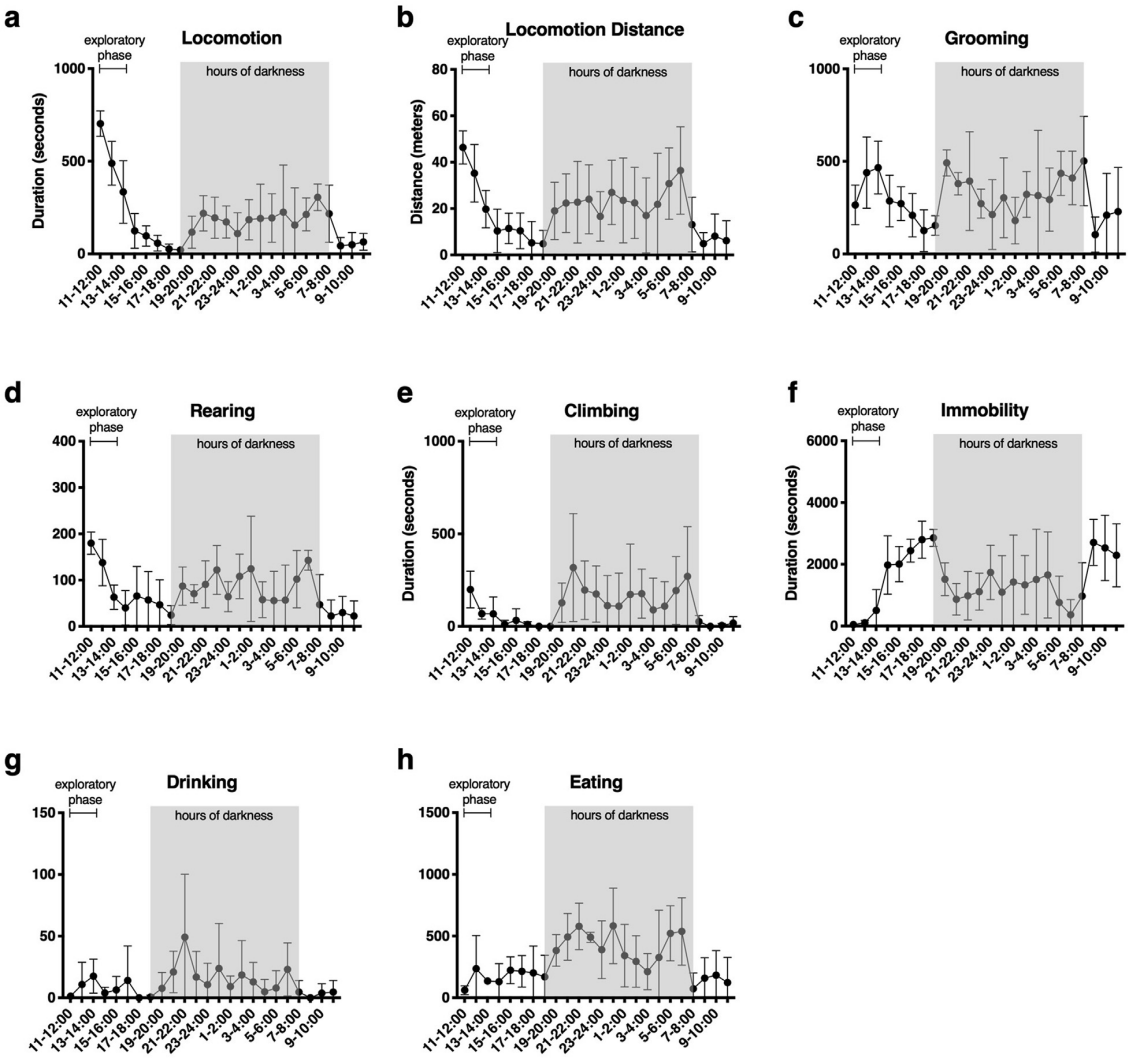
LABORAS recording over 24 h in naive animals. Duration of activity, or distance travelled, of each individual behaviour were recorded over a 24-h LABORAS session in naive healthy animals from 11:00–11:00. Note different scales of y axes. n = 4.95% CI shown.

Four non-operated (naive) animals were assessed over a 24-h period by LABORAS in order to establish the activity patterns of naive animals over time in our unit. Fig. 2 shows the activity profile for a 24-h period from 11:00 until 11:00 the next day, with each panel (a-h) showing the activity of one specific behaviour. High activity levels were observed in the first 3 h (exploratory phase). Activity levels dropped from 14:00–19:00 in keeping with known low murine activity during daylight hours. After 19:00, which marks the beginning of the dark phase in the animal unit, overall activity in all behaviours increased until lights were turned on again at 7:00 the next day. Finally, there was a reduction of activity in the hours of light in the morning (7:00–11:00). There was high variation in activity between individual animals as reflected by the large standard deviation.

### 3.3 Inflammatory arthritis caused a reduction in all activities by LABORAS

**Fig. 3:**
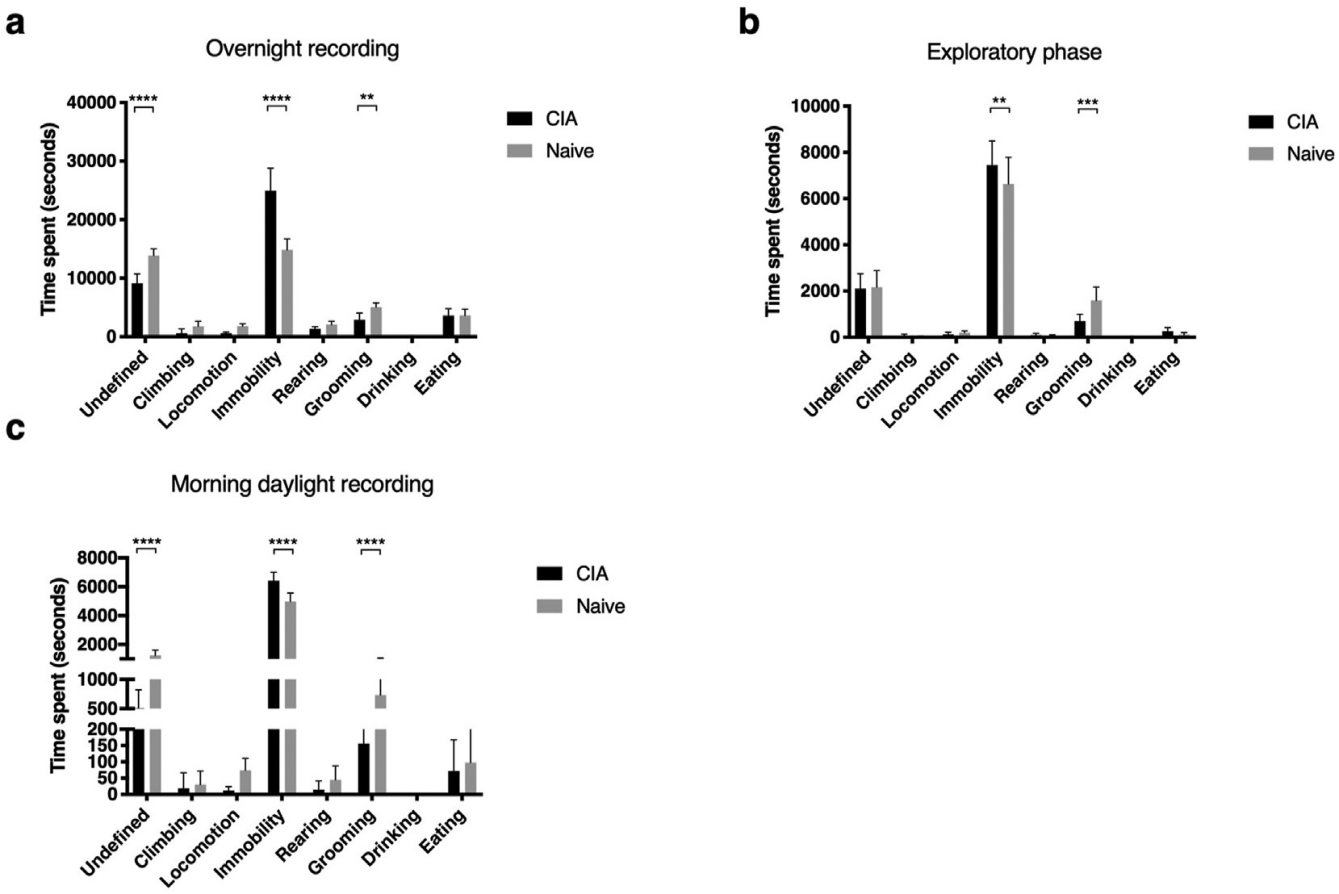
LABORAS recording in CIA animals. (a) 12-h recording from 19:00–7:00 (hours of darkness) of CIA and naive animals. (b) Exploratory phase 3-h recording of activity in CIA and naive animals from 15:00–18:00. (c) Daylight recording from 7:00–9:00. Two-way ANOVA with significances after Bonferroni multiple comparisons test shown. n = 10 each group. ∗∗p < 0.01, ∗∗∗p < 0.001, ∗∗∗∗p < 0.0001, 95% CI shown.

A positive control experiment was conducted in mice with collagen-induced arthritis (CIA) to assess the ability of LABORAS to detect painful behaviour based on activity levels. Animals with established active collagen-induced arthritis (CIA) in at least one hind paw (n = 10) were compared with naive control animals. During hours of darkness (19:00–7:00 h) CIA animals spent significantly more time inactive (‘immobility’), less time in ‘undefined’ and ‘grooming’ activities than their non-arthritic control group (naïve) (Fig. 3a). Other activities, were not significantly changed. The pattern of activity change was similar when considering the ‘exploratory phase’ or ‘morning daylight’ recordings Fig. 3b and c). In conclusion, pain behaviour was readily detectable in mice with CIA. The dark period was selected as the most sensitive period to measure changes in behaviour for subsequent studies.

### 3.4 Post-operative pain behaviour post PMX

**Fig. 4:**
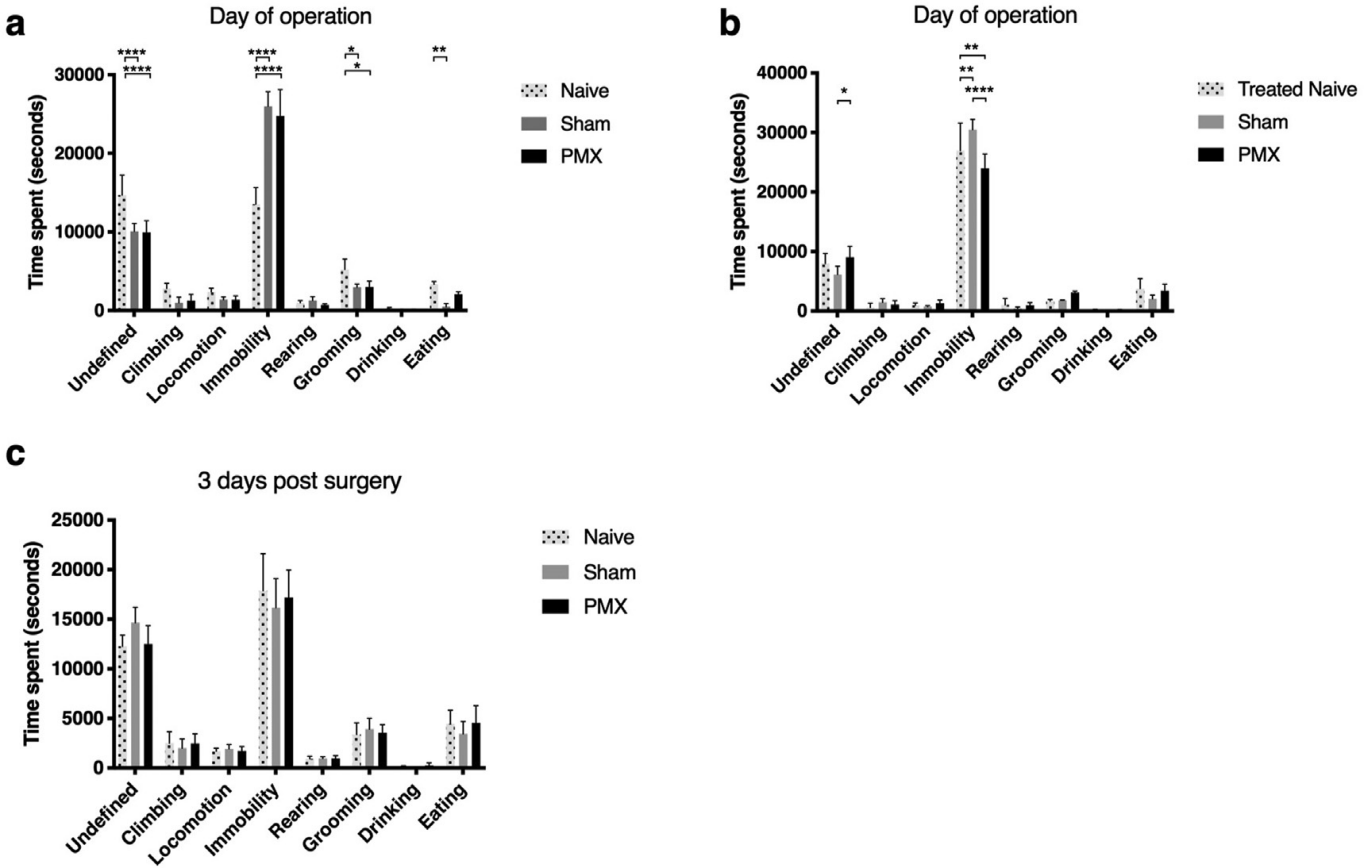
LABORAS in post-surgical animals compared with naive (un-operated) animals from 19:00–7:00. (a) 12-h recording of OA- and sham-operated as well as naive mice starting on the day of operation with naive animals not having received anaesthetic nor analgesia. (b) 12-h recording of OA- and sham-operated as well as naive mice on day of operation with naive animals having received matched anaesthetic and analgesia. (c) Same animals as (a) 3 days after surgery. Ordinary two-way ANOVA with Tukey's multiple comparisons test. n = 4 each group. ∗p < 0.05, ∗∗p < 0.01, ∗∗∗∗p < 0.0001.95% CI shown.

We first assessed the immediate post-operative phase of pain using LABORAS. We measured post-op pain in PMX and sham-operated animals whose surgery had taken place between 9:00 and 11:00 a.m. in the morning, and compared activities with naive (non-operated) mice. Mice were assessed from 19:00–7:00 starting on the day of surgery or three days after surgery. In the first experiment, naive animals did not receive analgesia or anaesthetic treatment (Fig. 4a). There were significant differences in ‘undefined’, ‘immobility’, ‘grooming’, and ‘eating’. However, when analgesia and anaesthetic were controlled for in the naive animals, these differences were lost and surgical groups did not display increased pain behaviour relative to naïve animals (Fig. 4b). Differences between the three groups had disappeared completely by three days after surgery (Fig. 4c).

### 3.5 LABORAS was unable to detect significant behavioural differences over the course of OA

**Fig. 5:**
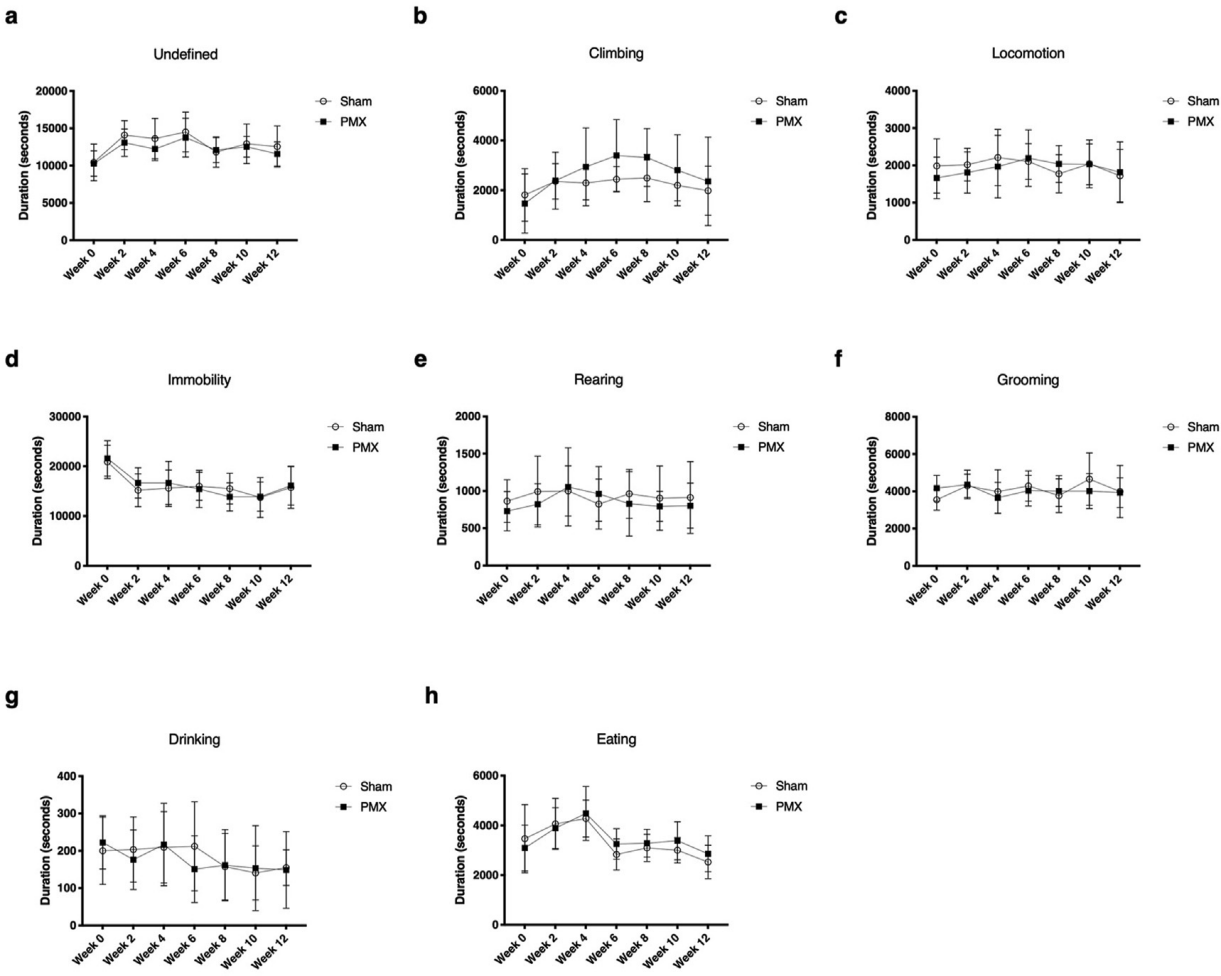
Sum of each activity of weekly LABORAS recordings overnight after OA- or sham surgery from 19:00–6:00 from the day of the operation until 12 weeks post-op. Multiple t-tests with a Bonferroni correction for multiple comparisons yielded no statistically significant results. n = 10 Sham, n = 10 PMX. 95% CI shown.

**Supplementary Fig. 1:**
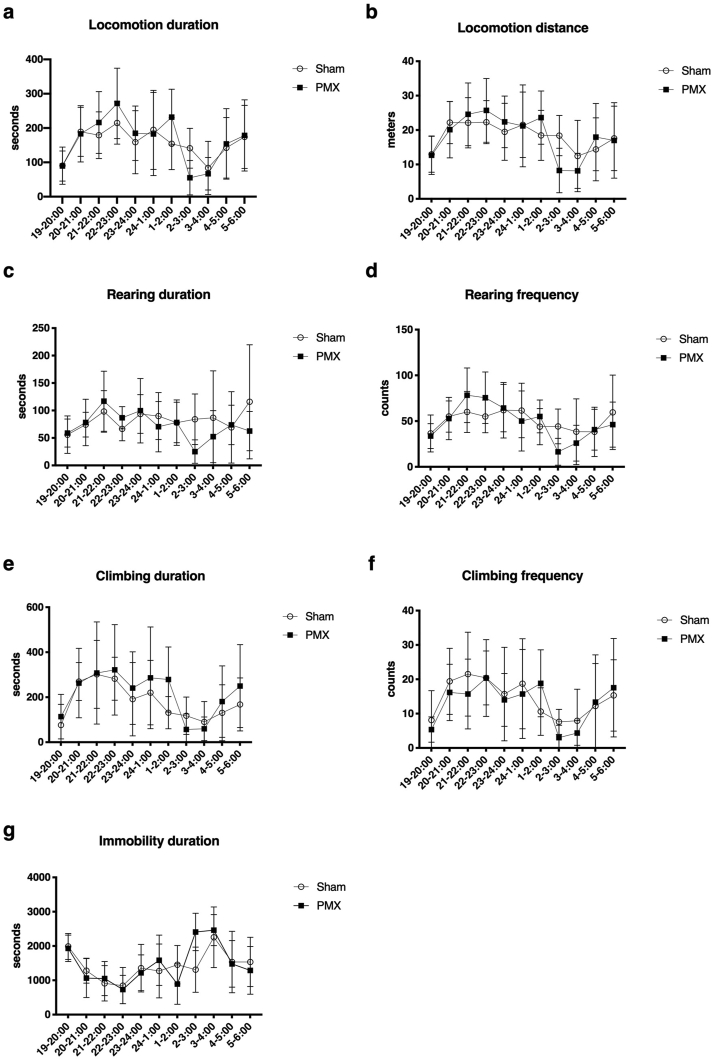
Hourly LABORAS recordings 12 weeks after surgery of individual activities overnight from 19:00–6:00 from the same animals as in Fig. 5. Average of individual activities (locomotion, rearing, climbing and immobility) shown. Data did not fit statistical assumptions and therefore was analysed using a *t*-test with a Bonferroni post hoc for multiple comparisons with no statistically significant differences found. Sham n = 10, PMX n = 10.95% CI shown.

We measured behavioural activity over the subsequent 12 weeks in sham- and PMX-operated animals using LABORAS. Fig. 5(a–h) shows bi-weekly recordings over a full overnight recording (19:00–6:00) until 12 weeks after surgery, the time at which animals exhibited robust painful behaviour by static incapacitance. No significant differences within groups over the course of OA development were detected using LABORAS. The 12 week data were also analysed further by considering the results for each hour separately and by assessing the distance travelled and frequency of initiations of ‘rearing’ and ‘climbing’ rather than total duration. Similar to Fig. 5, no statistically significant changes were observed (Supplementary Fig. 1a–h).

LABORAS was unable to detect significant behavioural differences 12 weeks after OA surgery in mice ‘unacclimatised’ to LABORAS.

**Fig. 6:**
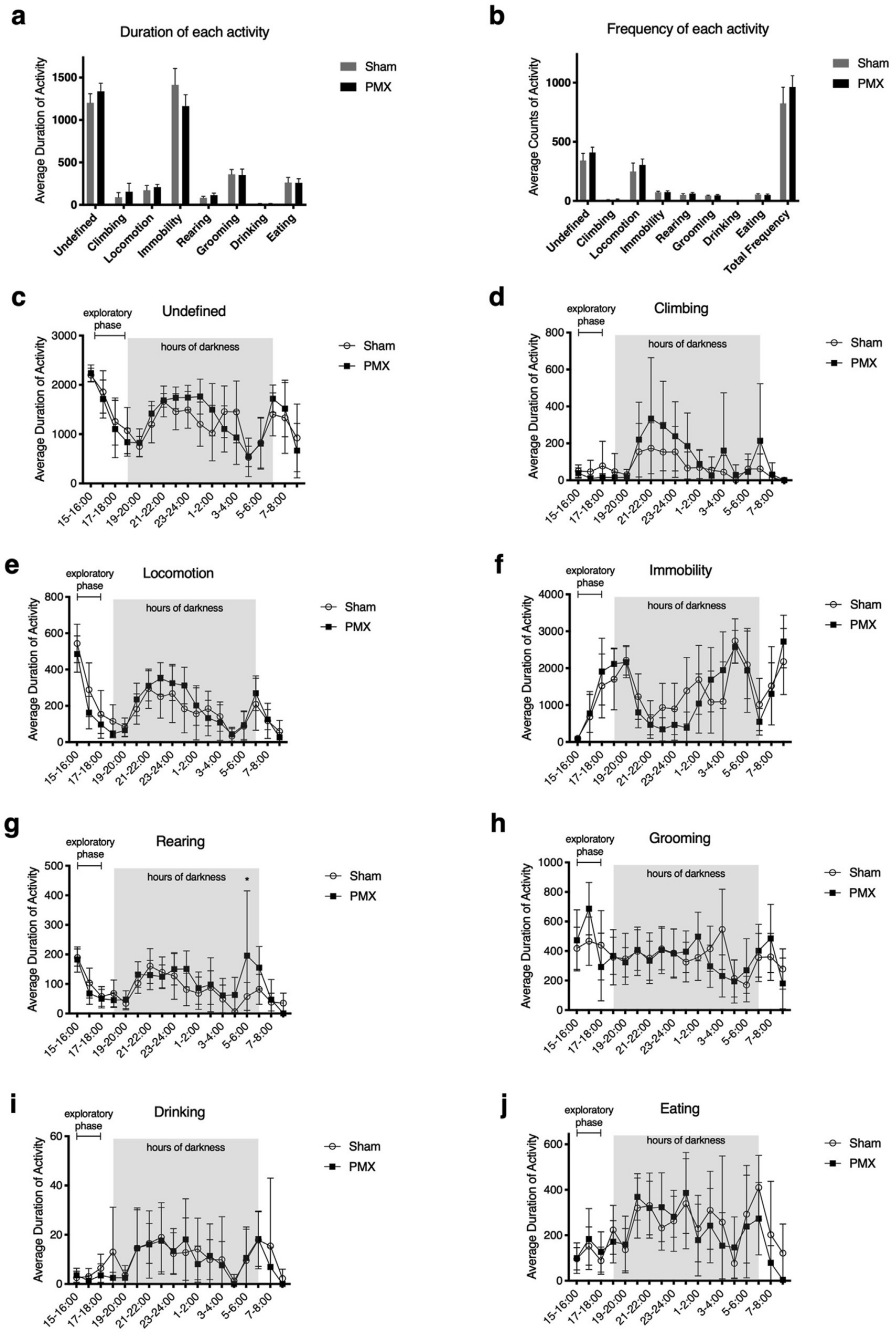
One-off LABORAS (non-acclimatised) recording 12 weeks after PMX or sham surgery. (a) Average duration of activity across 12 h (19:00–7:00) and (b) frequency of initiation of behaviours over the same timeframe. Statistical analysis in (a) and (b) used multiple t-tests with a Bonferroni test for multiple comparisons and yielded no statistically significant differences. (c) Breakdown of each individual behaviour over the full 12 h recording period. Data did not fit statistical assumptions and therefore was analysed using a *t*-test with a Bonferroni post hoc for multiple comparisons with no statistically significant differences found. There were some missing data points (n = 10/group from 15:00–3:00, and n = 8/group from 3:00–9:00 due to equipment failure over one night). n = 10/group. 95% CI shown.

**Fig. 7:**
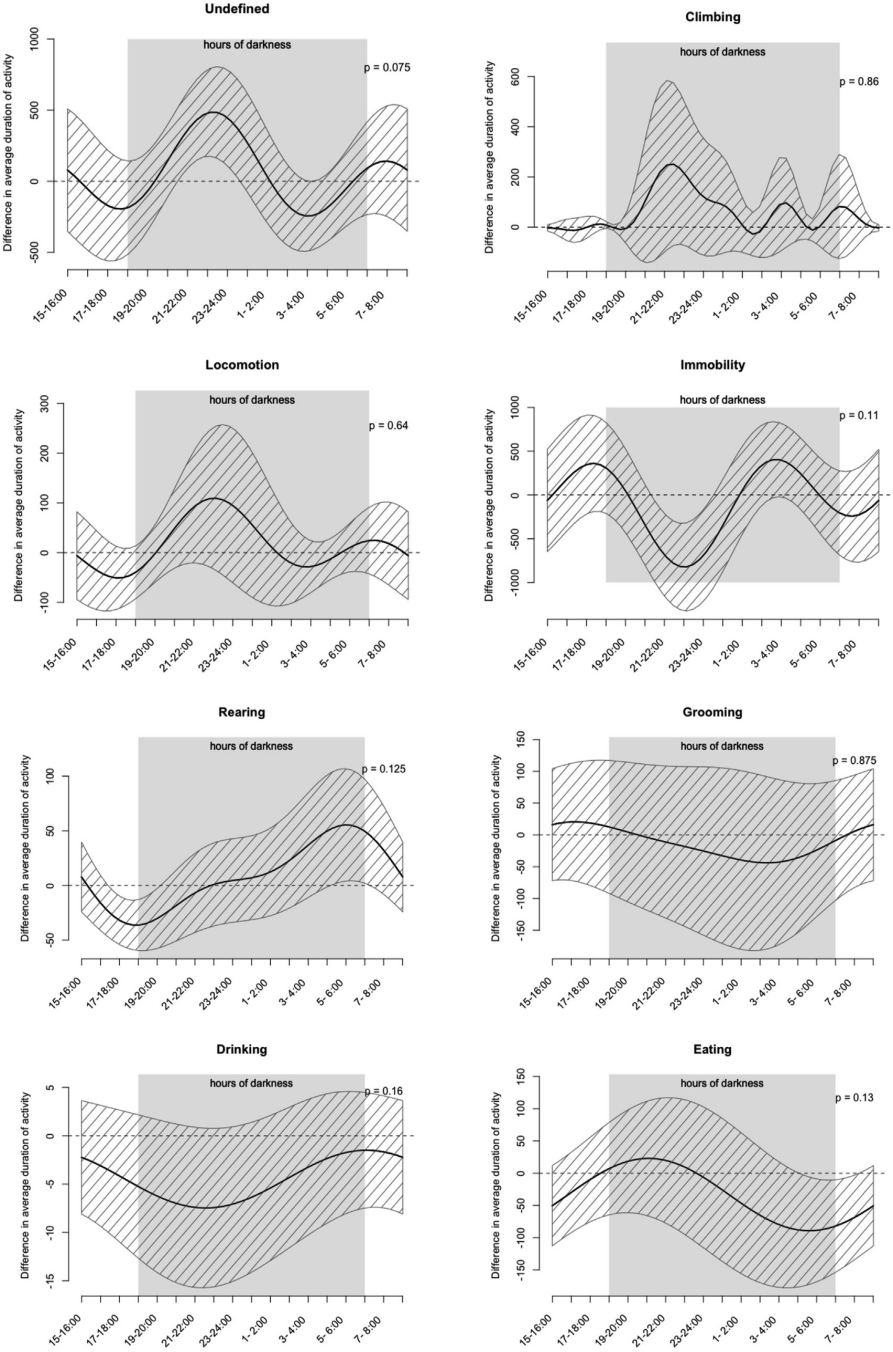
Estimated difference in duration between OA- and sham surgery for each of the measured behaviours, calculated via functional regression analysis of the 12 week one-off LABORAS recording. The dashed area shows the 95% confidence interval on the estimate. *P*-values were calculated using the maximum F statistic, with a distribution under the null hypothesis of zero difference between OA- and sham surgery generated by 1000 permutations. n = 8/group.

In a final experiment we assessed whether we lost sensitivity by acclimatizing the animals to their environment. We wanted to test whether we could detect significant differences in behaviour in animals 12 weeks after surgery that had never been acclimatised to LABORAS previously and which were only tested once at a time of established pain behaviour. Fig. 6a shows the average duration of activity over a 12-h recording, with Fig. 7b indicating the average frequency of each initiated behaviour. Fig. 7c–j shows the breakdown of every behaviour over the course of the entire recording period. There were no significant differences in the overall duration or frequency of behaviours between groups.

It was possible that the lack of significant effects when testing individual time points was due to the reduction in power from the multiple testing burden. To increase power, and to combine information across time points, we carried out a functional regression analysis to model differences in behaviour between PMX and sham groups across the time-course of the experiment. This allowed us to estimate a confidence interval of plausible effects of the PMX treatment over time for each of the behaviours (Fig. 7) In general we found very wide confidence intervals, suggesting that variation in the behavioural time-courses between mice made accurate estimation of the treatment effect difficult. We calculated p-values against the null hypothesis that PMX had no effect at any time point, and for each behaviour the data was compatible with this null hypothesis (p > 0.05).

## 4 Discussion

We have performed a comparison of two measures of spontaneous pain behaviour in a surgical model of murine OA. Static incapacitance measures the percentage weight distribution through the hind limbs, and as such offers a direct behavioural measure of pain at rest. Similar to human pain behaviour, animals off-load the affected painful joint and this can be expressed as the percentage of weight borne by the operated compared with the non-operated limb. By static incapacitance, we confirm two phases of painful behaviour: one in the immediate post-operative period that occurs in both sham and joint-destabilised animals, and a second late change in behaviour, arising from 8 weeks after surgery in both groups, but only statistically significant in the destabilised group at 12 weeks. The time of onset of pain behaviour is remarkably constant; occurring at 10–11 weeks following DMM surgery [9,26] and 8 weeks following PMX [27]. This appears to be in line with the more rapid cartilage damage seen after PMX, with chondropathy score appearing to predict onset of disease [26].

This type of pain assessment is prone to observer bias as the observer has to make a judgement about when the animals are standing appropriately and when to take the measurement. To mitigate bias it is essential to blind the observer to treatment (sham or OA surgery) and if possible to the time post surgery. The latter is rarely feasible in practice and was not performed in this study. This might account for why we observed a modest, non-progressive weight bearing asymmetry in sham-operated mice from 8 weeks following surgery. In principle, a second observer could reduce bias. However, one is usually limited by the number of tests each mouse can be subjected to, a desire to reduce unnecessary handling, and the number of individuals who handle the mice. In this study the observer was blinded to treatment and un-blinding only occurred after pain behaviour data had been gathered. Other confounding factors were also controlled for; all assessments were performed at 7am in an otherwise empty animal behavioural suite; the observations were made by a single female operative (IvL); mice were fully acclimatised to handling and the Linton apparatus prior to surgery; and additional care was taken not to stress the mice by avoiding tail lifting and scruffing. Once established this method of pain behaviour assessment is rapid, typically taking around 20 min to test a cage of 5 animals.

A second method of assessing spontaneous painful behaviour in mice was performed by LABORAS. This method has the advantage that objective behavioural assessment of the animals can occur without observer-dependent bias or interference. As the measurements are occurring overnight there are few other environmental confounding factors to control for. In addition, it measures pain behaviour on activity, which is theoretically more sensitive than standing pain. However, in this study, following PMX, we were not able to demonstrate pain behaviour using LABORAS, even though we were able to detect significant differences in behaviour in an inflammatory model of arthritis. Although there initially appeared to be post-operative changes in behaviour, when the naive (non-operated) mice received the anaesthetic and analgesia, this effect was lost.

Some OA pain studies have, however, been able to find significant differences using LABORAS, including one from our group [9,13,20]. Each of these used a different outcome measure to demonstrate a change in pain behaviour. Therefore, in the current study we examined multiple outcome measures: (i) individual activities or activity as a whole over the light/dark periods, (ii) activities over a 12 week time course, with assessments every two weeks (iii) number of initiations of a given movement rather than the time spent on a particular activity (iv) activity at 12 weeks post surgery in animals that were unacclimatised to LABORAS. None of these showed changes in pain behaviour that reached statistical significance although the high variance between mice might have masked a meaningful effect. We also chose to include “undefined” behaviour, which, in this study, constituted approximately 25% of total mouse activity. Whether this is higher than usual for this type of study is difficult to gauge, as not all published studies have reported it. It was nonetheless interesting that “undefined behaviour” was significantly reduced in mice with CIA. As pain in OA is insidious and modest grade, and the disease is not characterised by systemic inflammation, our results suggest that LABORAS is more sensitive at picking up pain associated with systemic inflammation and malaise.

To what extent are our findings simply related to inadequate power? This is certainly likely to be the case for incapacitance testing where evident trends in behaviour are apparent from 8 weeks but these only reached statistical significance at 12 weeks (Fig. 1). For this particular study we had a larger cohort in the incapacitance study (n = 15 PMX) than the LABORAS study. These typical numbers are required to make statistically robust conclusions. Given the large variability of individual mouse activity in LABORAS, we were potentially underpowered. However, we saw no trends in pain behaviour differences using LABORAS. LABORAS testing is also very labour intensive. Mice require single housing for the 15 h study period and most groups have only 2 or 4 cages. To perform this study with 10 mice in each group, the LABORAS apparatus was used most nights for the 12 weeks of the study. Increasing numbers would not have been a practical consideration.

An additional observation made in this study was that the exploratory phase (first 3 h) was the least sensitive period to examine pain behaviour in inflammatory arthritis. This is in contrast to findings of Cobos and colleagues [28], where the first hour of activity on a treadmill was sufficient to measure spontaneous painful behaviour in mice.

There is no doubt that spontaneous pain behaviour is challenging to measure in non-inflammatory models of OA, especially in mice. This is partly related to their innate instinct to hide pain behaviour to protect from predation [29]. However, spontaneous pain that we measure by incapacitance testing is robust and mirrors the clinical situation, being insidious and progressive; murine pain behaviour comes on relatively late in disease at a time when there is established structural change in the joint and it is sensitive to neutralisation by NGF similar to human disease [18,30]. Even though LABORAS is potentially a more refined procedure as it does not confine the animals, our data support the use of incapacitance testing, rather than LABORAS, for assessing spontaneous pain behaviour in surgically induced murine OA.

## Author contributions

I.v.L. and T.V. designed the studies. J.Z. and I. v.L. conducted the mouse surgeries and I. v.L. conducted all behavioural studies and analyses. I.H. conducted the CIA-induction under R.W. guidance. L.J. conducted the statistical analysis using R and advised on statistical tests throughout the project. I. v.L. and T.V. wrote the manuscript with revisions included from R.W. and L.J. Responsibility for the integrity of work as a whole lies with I. v.L. and T.V.

## Role of funding source

This work was supported by 10.13039/501100002283ARUK Grants 20,205 and 21,621 and 10.13039/100004440Wellcome Trust/10.13039/501100000288Royal Society grant 208750/Z/17/Z, as well as the Kennedy Prize Studentship Grant MSP 141503.

## Declaration of competing interest

N/A.
